# PAPP‐A functions as a tumor suppressor and is downregulated in renal cell carcinoma

**DOI:** 10.1002/2211-5463.13156

**Published:** 2021-05-02

**Authors:** Yanxin Lu, Shi Li, Tongyu Wang, Ximian Liao, Longyi Mao, Zesong Li

**Affiliations:** ^1^ Guangdong Key Laboratory of Systems Biology and Synthetic Biology for Urogenital Tumors Shenzhen Second People's Hospital First Affiliated Hospital of Shenzhen University China; ^2^ Zhuhai Campus of Zunyi Medical University China; ^3^ Shenzhen Institute of Advanced Technology Chinese Academy of Science Shenzhen China; ^4^ Shenzhen Key Laboratory of Genitourinary Tumor Shenzhen Second People's Hospital First Affiliated Hospital of Shenzhen University China

**Keywords:** invasion, migration, pregnancy‐associated plasma protein A, proliferation, renal cell carcinoma, tumor suppressor

## Abstract

Pregnancy‐associated plasma protein A (PAPP‐A) is a proteolytic enzyme produced by the placenta. The expression and role of PAPP‐A in renal cell carcinoma (RCC) remain elusive. The aim of this study was to investigate the role and the molecular mechanisms of PAPP‐A in RCC. Initially, we evaluated the expression of PAPP‐A in samples from patients with RCC and cell lines by quantitative PCR, western blot and immunohistochemical staining, and examined the role of PAPP‐A in RCC cells by cell viability, colony formation and Transwell assays. Next, we investigated the molecular mechanisms regulating the tumor suppressor function of PAPP‐A. Our results demonstrated that PAPP‐A is expressed at low levels in RCC tissues and cells. Clinical data analysis revealed a significant correlation between PAPP‐A expression and RCC‐related death (*P* < 0.0115). Overexpression of PAPP‐A inhibited viability, proliferation, migration and invasion of RCC cells. Furthermore, PAPP‐A overexpression significantly increased phosphorylation of c‐Jun N‐terminal kinase and decreased the expression of cyclin D1, phosphorylated glycogen synthase kinase‐3β and β‐catenin. This study is the first to report that downregulation of PAPP‐A is associated with poor prognosis in patients with RCC. In conclusion, PAPP‐A may serve as a novel prognostic marker and potentially as a therapeutic target in patients with RCC.

## Graphical abstract

Here, we investigated the expression and function of pregnancy‐associated plasma protein A (PAPP‐A) in renal cell carcinoma (RCC). PAPP‐A is significantly downregulated in RCC and is associated with poor prognosis in patients with RCC. Overexpression of PAPP‐A inhibited viability, proliferation, migration and invasion of RCC cells. Furthermore, PAPP‐A overexpression significantly increased phosphorylation of c‐Jun N‐terminal kinase and decreased the expression of cyclin D1, phosphorylated glycogen synthase kinase‐3β and β‐catenin.

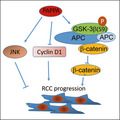

**Abbreviations**
CCK‐8: Cell Counting Kit‐8
ccRCC: clear cell renal cell carcinoma
EdU: 5‐ethynyl‐2' ‐deoxyuridine
ER: Estrogen Receptor
GSK‐3β: glycogen synthase kinase‐3β
IGF: insulin‐like growth factor
IHC: immunohistochemistry
JNK: c‐Jun N‐terminal kinase
LOH: loss of heterozygosity
MCP‐1: monocyte chemoattractant protein‐1
p‐: phosphorylated
PAPP‐A: pregnancy‐associated plasma protein A
qPCR: quantitative PCR
RCC: renal cell carcinoma
qRT‐PCR: quantitative RT‐PCR
SD: standard deviation
PI3K: phosphatidylinositol 3 kinase
SAPK/JNK: Stress Activated Protein Kinase/Jun N‐Terminal Kinase
TCGA: The Cancer Genome Atlas

Renal cell carcinoma (RCC) comprises 2% to 3% of all adult malignancies; RCC has had a high incidence rate in recent years and accounts for ~80% of primary renal malignancies [1]. Clear cell RCC (ccRCC) accounts for about 80% of RCCs and is the most aggressive subtype of RCC. Partial nephrectomy is the most effective therapeutic method for early and local RCC. However, up to 30% of patients experience development of metastases after surgery [2]. The mechanism of RCC formation and progression remains unclear. Therefore, exploring new targets associated with tumorigenesis may improve the potential therapeutic strategies and the therapeutic response of RCC.

Pregnancy‐associated plasma protein A (PAPP‐A), also named PAPP‐A lysine‐1, was first reported as a placental antigen with a high concentration in the plasma of pregnant women [3]. PAPP‐A is a zinc‐binding metalloproteinase protein and an activator of the insulin‐like growth factor (IGF) pathway [4]. PAPP‐A significantly increases MCP‐1, tumor necrosis factor‐α, and interleukin‐6 expression through activating the IGF‐I/PI3K/Akt signaling pathway in macrophages [5]. PAPP‐A is involved in atherosclerosis and plaque rupture progression, and acts as an inflammatory biomarker. Subsequent studies found that PAPP‐A is overexpressed in a variety of tumors, such as breast cancer, ovarian cancer and Ewing sarcoma, and that inhibition of PAPP‐A expression suppresses tumor growth and invasion [6, 7, 8]. Overexpression of PAPP‐A in ovarian cancer cell lines triggers tumorigenesis and cellular invasion [9], and targeting PAPP‐A using a monoclonal antibody reverses platinum resistance in ovarian cancer [8]. PAPP‐A is ubiquitously expressed in a variety of normal tissues and is most abundantly expressed in the kidney and bone [10, 11]. However, the expression level and function of PAPP‐A in ccRCC are still elusive.

In this study, we investigated the expression levels and roles of PAPP‐A in ccRCC. In contrast with the PAPP‐A expression pattern in the other types of tumors, the results of quantitative PCR (qPCR), western blot and immunohistochemistry (IHC) assays showed that PAPP‐A was low expressed in ccRCC tissues and cell lines compared with paracarcinoma tissue and normal cells. Kaplan–Meier survival analysis showed that PAPP‐A was a factor indicating the survival of patients with ccRCC. Furthermore, overexpression of PAPP‐A in ccRCC cells suppressed cell viability, proliferation, migration, and invasion via the c‐Jun N‐terminal kinase (JNK) and Wnt–β‐catenin pathways.

## Materials and methods

### Tissue samples

This study included 95 samples from patients diagnosed with ccRCC. Renal cancer tissues and paired paracarcinoma renal tissues were obtained from each patient and stored at −80 °C. Written informed consent was provided by the patients and their families for the use of tissue specimens. This study was approved by the Ethics Committee of Shenzhen Second People's Hospital (Shenzhen, China). The study methodologies conformed to the standards set by the Declaration of Helsinki. The ccRCC tissue microarray (HKid‐CRC180Sur‐01) was purchased from Shanghai Outdo Biotech Co., Ltd (Shanghai, China). The RNA expression data of PAPP‐A were profiled based on normalized RNA sequencing expression datasets of ccRCC in The Cancer Genome Atlas (TCGA) database (TCGA_KIRC_exp_HiSeqV2‐2015‐02‐24) from the University of California Santa Cruz (UCSC) Xena Browser (https://xenabrowser.net/datapages/).

### Cell culture and transfection

The human ccRCC cell lines OS‐RC‐2, 786‐O and 769‐P were purchased from the American Type Culture Collection (Manassas, VA, USA). OS‐RC‐2, 786‐O and 769‐P cells were cultured in MEM (Gibco; Thermo Fisher Scientific, Inc. Shanghai, China) or RPMI 1640 (Gibco; Thermo Fisher Scientific, Inc.) with 10% FBS (Gibco; Thermo Fisher Scientific. Inc.), 100 U·mL^−1^ penicillin (Gibco; Thermo Fisher Scientific, Inc.) and 100 µg·mL^−1^ streptomycin (Gibco; Thermo Fisher Scientific, Inc.). The human renal proximal small tube cell line (HK‐2), as a control cell line, was cultured in keratinocyte serum‐free medium with 0.05 mg·mL^−1^ bovine pituitary extract and 5 ng·mL^−1^ human recombinant epidermal growth factor. Cells were grown at 37 °C in a 5% CO_2_ atmosphere.

For transfection, the cells were seeded in six‐well plates at 70% confluence and transfected with pCMV6‐Entry‐PAPP‐A vector or pCMV6‐Entry vector using Lipofectamine 3000 (Invitrogen, Carlsbad, CA, USA) according to the manufacturer’s instructions. The eukaryotic expression plasmids pCMV6‐Entry‐PAPP‐A and pCMV6‐Entry were purchased from OriGene [catalog (cat) no. RC209767; Rockville, MD, USA].

### Real‐time quantitative RT‐PCR

Real‐time quantitative RT‐PCR (qRT‐PCR) was carried out as described previously [12]. Total RNA was extracted using TRIzol reagent (Invitrogen; Thermo Fisher Scientific, Inc.). cDNA was transcribed from total RNA using PrimeScript™ RT reagent with gDNA Eraser (Takara Bio, Inc., Otsu, Japan) according to the manufacturer’s protocol. Real‐time qPCR was performed using a SYBR Premix Pro Taq HS qPCR kit (Accurate Bio, Inc., Hunan, China) with the following cycling conditions: initial denaturation at 95 °C for 5 min, followed by 40 cycles of denaturation at 95 °C for 30 s and annealing at 60 °C for 30 s. The primer sequences were as follows: PAPP‐A, forward 5′‐ACAAAGACCCACGCTACTTTTT‐3′, reverse 5′‐CATGAACTGCCCATCATAGGTG‐3′; and ACTB, forward 5′‐TGAAGATCAAGATCATTGCTCCTC‐3′, reverse 5′‐AACTAAGTCATAGTCCGCCTAGAAG‐3′. Each sample was analyzed in triplicate using the ABI 7300 Real‐Time PCR system (Applied Biosystems; Thermo Fisher Scientific, Inc.). The relative expression levels of PAPP‐A were calculated using the 2^−ΔΔCq^ method as previously described [13].

### Western blot analysis

We followed the methods of Xia *et al*. [12]. Proteins from the cells were extracted using radio immunoprecipitation assay lysis buffer containing 1% protease inhibitor cocktail (Sigma‐Aldrich, Merck KGaA, Shanghai, China). Protein concentration was quantified using the bicinchoninic acid assay kit (Pierce; Thermo Fisher Scientific, Inc.). Proteins were separated by 10% SDS/PAGE and transferred onto poly(vinylidene difluoride) membranes (EMD Millipore, Billerica, MA, USA). Membranes were blocked in 5% skimmed milk at room temperature for 1 h and were incubated with the following primary antibodies diluted in TBST with 5% BSA (cat no. 4240GR100; NeoFROXX GmbH, Hesse, Germany) overnight at 4 °C: PAPP‐A (1 : 1000 dilution; cat no. sc‐365226; Santa Cruz Biotechnology, Inc., Dallas, TX, USA), β‐tubulin (1 : 5000 dilution; cat no. ab6046; Abcam, Cambridge, UK), phospho‐SAPK/JNK (Thr193/Tyr185; 1 : 1000 dilution; cat no. 9255; Cell Signaling Technology, Inc., Danvers, MA, USA), SAPK/JNK (1 : 1000 dilution; cat no. 9252; Cell Signaling Technology, Inc.), cyclin D1 (1 : 1000 dilution; cat no. 2978; Cell Signaling Technology, Inc.), phospho‐glycogen synthase kinase‐3β (phospho‐GSK‐3β; Ser9; 1 : 1000 dilution; cat no. 55585; Cell Signaling Technology, Inc.), GSK‐3β (3D10; 1 : 1000 dilution; cat no. 9832; Cell Signaling Technology, Inc.) and β‐catenin (1 : 1000 dilution; cat no. sc‐7963; Santa Cruz Biotechnology, Inc.). After being washed with TBST [20 mm Tris–HCl (pH 7.5), 150 mm NaCl, 0.1% Tween 20], the membranes were incubated with goat anti‐rabbit (1 : 1000 dilution; cat no. sc‐2004; Santa Cruz Biotechnology, Inc.) or horse anti‐mouse (1 : 1000 dilution; cat no. 7076P2; Cell Signaling Technology, Inc.) at room temperature for 1 h. Bands were visualized using an ECL kit (EMD Millipore) and detected by the Alliance Imaging system (UVITEC, Cambridge, UK).

### Immunohistochemical staining

A total of 112 RCC specimens and paracarcinoma tissues that were fixed in 10% formalin and paraffin embedded were purchased from Shanghai Outdo Biotech Co., Ltd. (HKid‐CRC180Sur‐01). The expression of PAPP‐A was determined through IHC. The sections were deparaffinized in xylene and then rehydrated in graded alcohols, and antigen was retrieved in 10 mm citrate buffer solution for 15 min using a microwave. The sections were inactivated by 3% H_2_O_2_, blocked with 5% goat serum for 30 min at room temperature and incubated with PAPP‐A antibody overnight at 4 °C. The sections were washed with PBS and incubated with secondary antibody labeled with peroxidase for 1 h at room temperature. Subsequently, sections were visualized with a 2,4‐diaminobutyric acid kit (Zhongshan Golden Bridge Biotechnology, Beijing, China), and the nucleus was counterstained with hematoxylin.

IHC was used to score specimens by the intensity (0, negative; 1, weak; 2, moderate; 3, strong) and percentage (1, ≤25%; 2, 26–50%; 3, 51–75%; 4, >76%) of positively stained cells. The IHC score was obtained by multiplying the intensity score by the score for the percentage of positively stained cells. A score <1 was considered negative expression, a score ≥1 and <5 was considered low expression, and a score ≥5 was considered high expression. Specimens were blindly evaluated by four pathologists to determine the IHC scores.

### Cell viability assay

Cell viability was assessed using the Cell Counting Kit‐8 (CCK‐8; Beyotime Institute of Biotechnology, Shanghai, China) assay. PAPP‐A‐ or control plasmid‐transfected cells were seeded in a 96‐well plate at 2 × 10^3^ cells per well. At 0, 24, 48, 72 and 96 h after transfection, cells were added with CCK‐8 reagent (10 µL in 100 µL medium for each well) and incubated at 37 °C for 2 h. Then the absorbance at 450 nm (*A*_450_ _nm_) was detected using a microplate reader (Bio‐Rad Laboratories Inc., Hercules, CA, USA). With regard to the colony formation assays, a total of 800 cells were seeded in a six‐well plate and cultured in RPMI 1640 (Gibco; Thermo Fisher Scientific, Inc.) with 10% FBS for 2 weeks. Cells were fixed with 4% paraformaldehyde for 20 min and then stained with 0.1% crystal violet for 5 min at room temperature. Images of the results were captured by using a camera. The number of colonies was counted using imagej [14]. Each experiment was performed in triplicate.

### Cell migration and invasion assays

Transwell assay was performed as described previously [12]. Cell migration was measured using 24‐well Transwell chambers (pore size, 8 µm; Corning Incorporated, Corning, NY, USA). In total, 5 × 10^4^ cells per well were seeded into the upper chamber in serum‐free medium. For the cell invasion assay, 5 × 10^4^ cells per well were seeded into the upper chamber, which was coated with Matrigel (1 : 8; 50 µL·well^−1^; BD Biosciences, San Jose, CA, USA). The lower chamber was filled with RPMI 1640 (Gibco; Thermo Fisher Scientific, Inc.) medium with 10% FBS. After 24 h, the cells in the upper chamber were removed with cotton swabs, and the cells in the bottom chamber were fixed with 4% paraformaldehyde for 15 min and stained using 0.1% crystal violet for 5 min at room temperature. Images migrated, and invaded cells were captured using an inverted microscope.

### Statistical analysis

The statistical software graphpad prism 8.0 (La Jolla, CA, USA) was used for all statistical analyses. All values are expressed as the mean ± standard deviation (SD). The differences between groups were assessed by *t*‐test for two groups or by one‐way ANOVA or two‐way ANOVA for more than two groups. For clinical characteristics analysis, the chi‐square test and Fisher’s exact test were used for statistical analyses. The association between PAPP‐A expression, evaluated by IHC, and survival time was analyzed using the log rank test and evaluated by drawing Kaplan–Meier curves. *P* < 0.05 was considered statistically significant.

## Results

### PAPP‐A expression is low in ccRCC

**Fig. 1:**
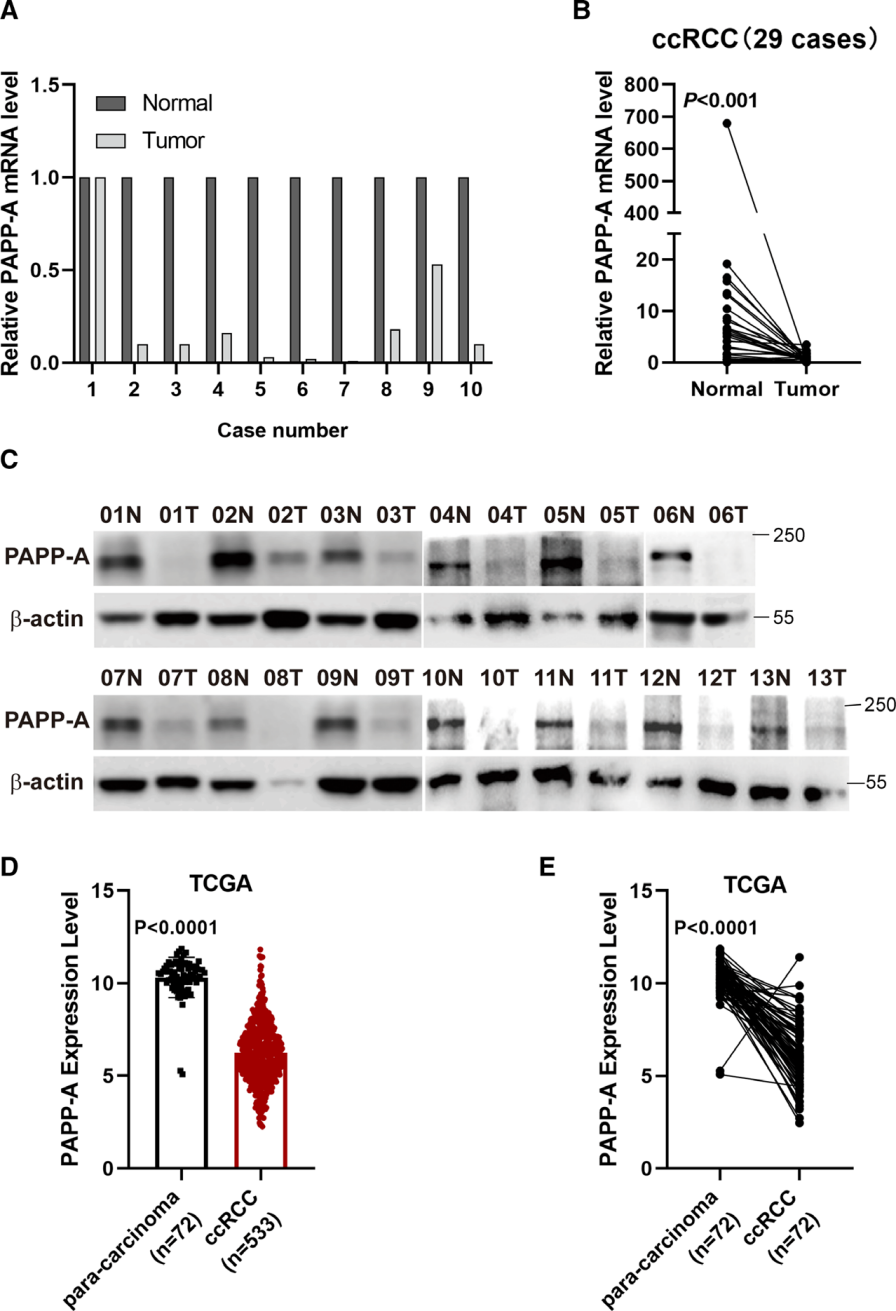
PAPP‐A expression is reduced in RCC tissue and cells. (A) Deep transcription sequencing analysis of 10 paired ccRCC tissues showed that PAPP‐A was expressed at low levels in tumor samples. (B) qPCR results showed that PAPP‐A mRNA was decreased in ccRCC tissue compared with normal tissue (*n* = 29). Mean ± SD is shown. *P* < 0.001 by Student’s *t*‐test. (C) Western blot results showed that PAPP‐A protein was expressed at low levels in ccRCC tissue. (D, E) TCGA analysis revealed that PAPP‐A was expressed at low levels in ccRCC samples (D: *n* = 72, paracarcinoma; *n* = 533, ccRCC; E: *n* = 72). Mean ± SD is shown. *P* < 0.0001 by Student’s *t*‐test.

The mRNA expression levels of PAPP‐A were found to be lower in ccRCC tissue than in corresponding healthy kidney tissue samples by deep transcription sequencing analysis of 10 paired ccRCC samples [13] (Fig. 1A). This finding is inconsistent with the PAPP‐A expression pattern in other kinds of tumors. To further investigate the expression of PAPP‐A in ccRCC, we detected the mRNA and protein levels of PAPP‐A using real‐time qPCR and western blot in 29 and 13 cases, respectively. As shown in Fig. 1B,C, PAPP‐A was silenced or expressed at lower levels in ccRCC tissues than in paracarcinoma tissues both at the mRNA and protein levels (Fig. 1B,C). An analysis of the TCGA database also revealed that PAPP‐A expression was significantly lower in ccRCC tissues than in paracarcinoma tissues (Fig. 1D,E).

### Correlations between PAPP‐A expression and clinical characteristics of patients with ccRCC

**Fig. 2:**
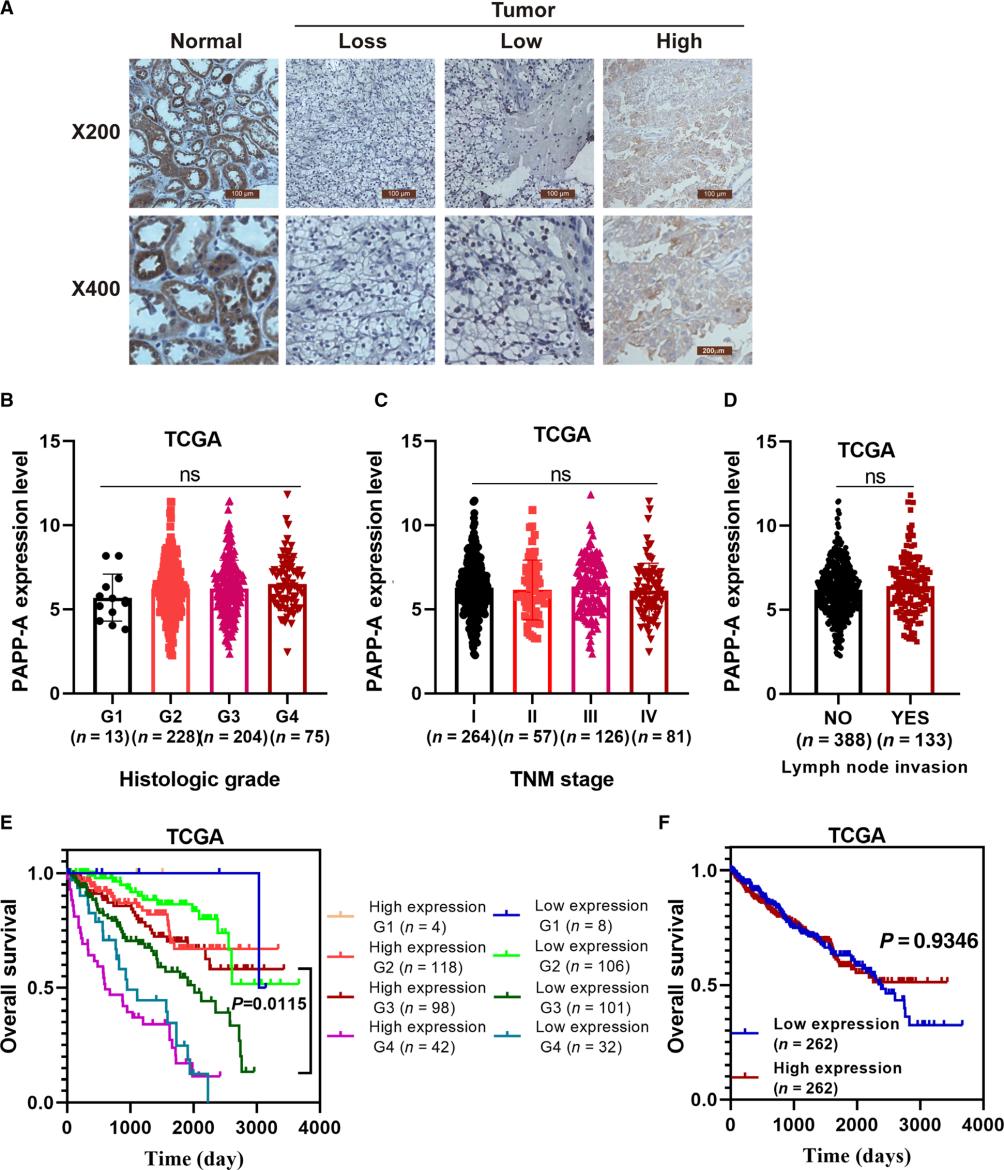
Relationship between PAPP‐A expression and prognosis of patients with ccRCC. (A) PAPP‐A expression was detected in ccRCC specimens and paired adjacent tissues using IHC. (B–D) Pathological correlation analysis results of PAPP‐A expression level and ccRCC histological grade (*n* = 13, G1; *n* = 228, G2; *n* = 204, G3; *n* = 75, G4), TNM stage (*n* = 264, stage I; *n* = 57, stage II; *n* = 126, stage III; *n* = 81, stage IV) and lymph node invasion (*n* = 388, no; *n* = 133, yes) using TCGA database. Mean ± SD is shown. (E) PAPP‐A expression is associated with the survival outcome of different histological grades for patients with ccRCC in the TCGA database (*n* = 4, G1 high; *n* = 8, G1 low; *n* = 118, G2 high; *n* = 106, G2 low; *n* = 98, G3 high; *n* = 101, G3 low; *n* = 42, G4 high; *n* = 32, G4 low). *P* = 0.0115 by log rank test. (F) TCGA analysis results showed PAPP‐A expression has no significant correction with the survival rate of the whole group ccRCC patients (*n* = 262). *P* = 0.6124 by log rank test. ns, not significant by Student’s *t*‐test.

To analyze the correlation of PAPP‐A expression level with patient outcome, we detected the expression of PAPP‐A using IHC in an ccRCC tissue microarray containing 56 paired samples. The patients were grouped into three categories by the expression level of PAPP‐A (negative, low and high; Fig. 2A). High expression of PAPP‐A was observed in paracarcinoma kidney tissues, and negative or low expression was observed in ccRCC tissues (*P* < 0.0001), as shown in Table 1. The expression of PAPP‐A was significantly associated with patient age (*P* = 0.0427) and tumor side (*P* = 0.0115). It is consistent with previous studies that ccRCC diagnosis is significantly associated with age [15]. However, there were no significant correlations identified between PAPP‐A expression and Fuhrman grade (*P* = 0.4122), TNM stage (*P* = 0.1882), sex (*P* = 0.8767) or tumor size (*P* = 0.3647; Table 1). Meanwhile, TCGA database analysis showed that the expression level of PAPP‐A had no significant differences between different grades (Fig. 2B), TNM stages (Fig. 2C) and lymph node metastasis status of RCC (Fig. 2D). We also analyzed the correction between PAPP‐A expression level and tumor side in more cases from TCGA database (Fig. S1A). The results showed there was no significant difference, which was consistent with the previous report [16].

**Table 1:** Association of PAPP‐A with clinicopathological characteristics in ccRCC.

Characteristics	Number of cases	PAPP‐A negative expression	PAPP‐A low expression	PAPP‐A high expression	χ^2^	*P* value
Type					75.83	<0.0001
Normal	35	0	0	35		
RCC	56	32	20	4		
Fuhrman grade					0.6667	0.4142
I + II	49	29	18	2		
III	7	3	2	2		
TNM stage					1.732	0.1882
I + II	47	26	16	2		
III + IV	4	1	1	2		
Sex					0.02409	0.8767
Male	31	18	9	4		
Female	25	14	11	0		
Age (years)					4.108	0.0427
≥60	32	22	9	1		
<60	24	10	11	3		
Size (cm)					0.8217	0.3647
≥4	46	25	17	4		
<4	10	7	3	0		
Tumor side					6.389	0.0115
Left kidney	22	8	13	1		
Right kidney	34	24	7	3		

Furthermore, we analyzed the TCGA database and discovered that the PAPP‐A expression level was differentially associated with prognosis in different histological grades of patients with RCC. TCGA database Kaplan–Meier analysis results showed that for RCC with grade 3, PAPP‐A expression was significantly inversely correlated with survival outcome, but for grades 1, 2, and 4 and whole group, there was no statistical difference (Fig. 2E,F). Each grade has typical pathological features and represents different grades of differentiation and tumor progression with different biological characteristics [17]. Therefore, there may have been various prognostic markers in different grades of ccRCC. The expression level of PAPP‐A is uniformly decreased regardless of the stage and grade of ccRCC, suggesting PAPP‐A may be involved in early carcinogenesis of ccRCC. In this regard, we analyzed the survival rate for localized cancer and metastatic cancer by PAPP‐A expression using TCGA database. The results showed that there was no statistical difference (Fig. S1B,C). Together, the results indicate that PAPP‐A is a potential prognostic marker for the overall survival of patients with grade 3 ccRCC.

### PAPP‐A inhibits the viability and proliferation of ccRCC cells

**Fig. 3:**
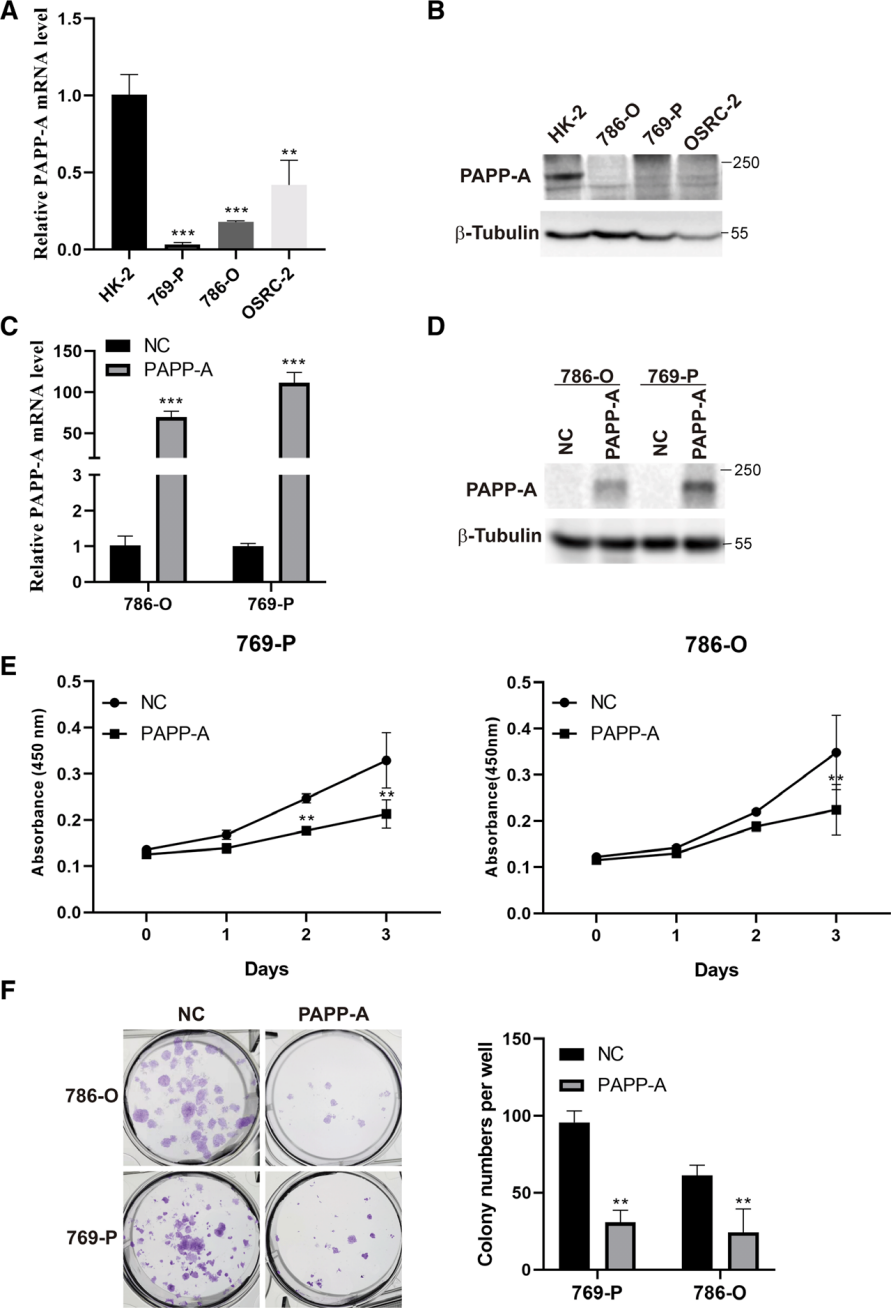
Ectopic expression of PAPP‐A suppresses ccRCC cell viability and proliferation. (A, B) Detection of PAPP‐A expression levels in human ccRCC cell lines and normal renal proximal tubular epithelial cells by qRT‐PCR and western blot. (C, D) Evaluation of the efficiency of PAPP‐A transfection using qRT‐PCR and western blot. (E) The effect of PAPP‐A on the viability of ccRCC cells in the CCK‐8 assay. (F) Colony formation assay shows the effect of PAPP‐A on the cell growth of ccRCC cells. *n* = 3. Mean ± SD is shown. **P < 0.01, ***P < 0.001 by Student’s *t*‐test.

The expression levels of PAPP‐A in ccRCC cell lines were determined by real‐time qPCR and western blot. The results revealed that the ccRCC cell lines 786‐O, 769‐P and OS‐RC‐2 had lower PAPP‐A mRNA and protein expression levels than the normal renal proximal tubular epithelial cell line HK‐2 (Fig. 3A,B). To determine the contribution of increased PAPP‐A expression on the tumorigenicity of ccRCC, we induced PAPP‐A overexpression in 786‐O and 769‐P ccRCC cell lines because these two cell lines exhibited the lowest mRNA and protein expression levels. ccRCC cells were transfected with a PAPP‐A eukaryotic expression construct or with empty vector as a control. The ectopic expression of exogenous PAPP‐A was confirmed by real‐time qPCR and western blot (Fig. 3C,D).

The effects of PAPP‐A on ccRCC cell viability and proliferation were assessed by CCK‐8 and cell colony formation assays. The CCK‐8 results showed that ectopic expression of PAPP‐A significantly reduced the cell viability of 786‐O and 769‐P cells in a time‐dependent manner (Fig. 3E). PAPP‐A‐overexpressing ccRCC cells exhibited smaller and fewer colonies than empty vector control cells in the cell colony formation assay (Fig. 3F). All of the results suggest that PAPP‐A inhibits ccRCC cell viability and proliferation.

### PAPP‐A suppresses the migration and invasion of ccRCC cells

**Fig. 4:**
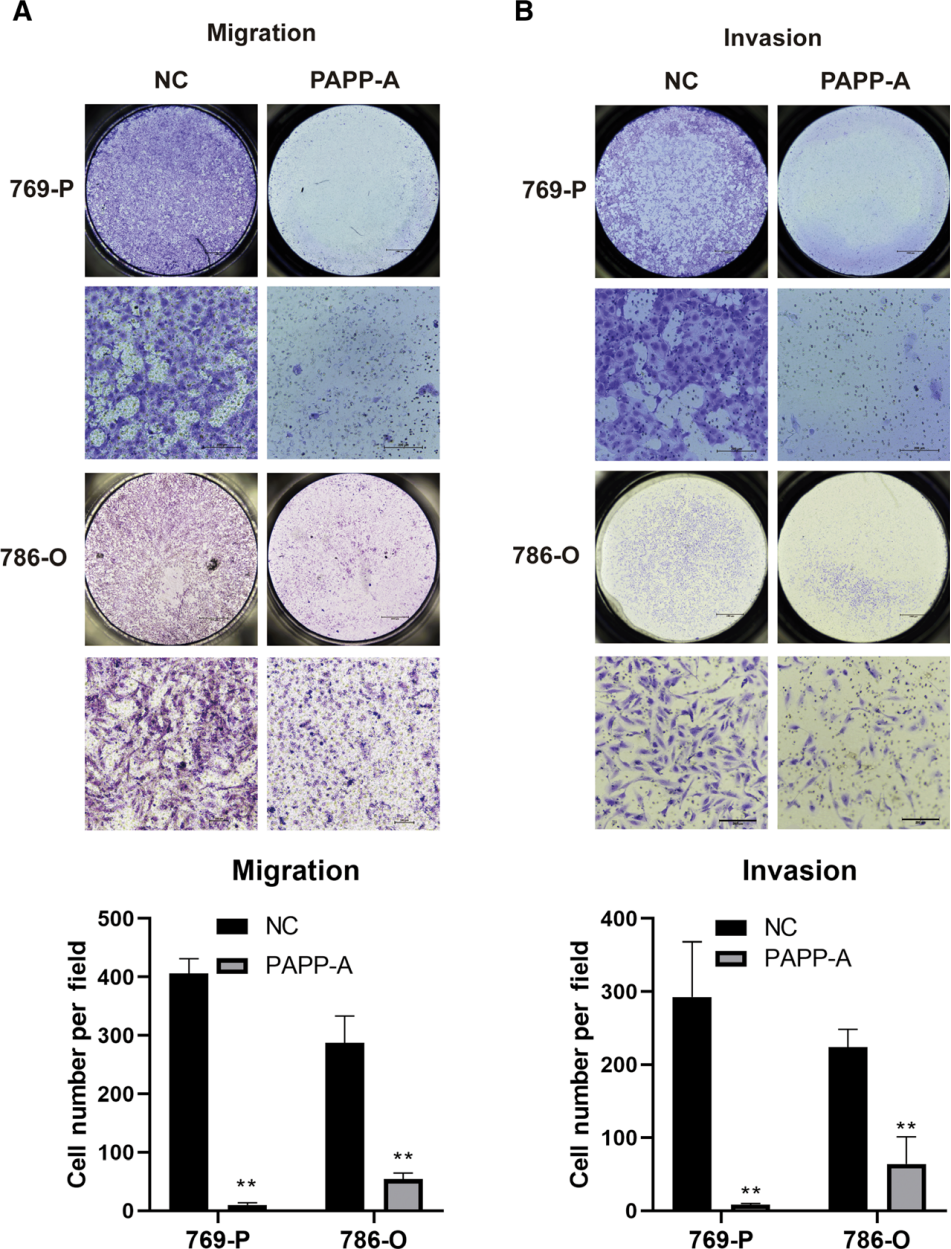
PAPP‐A inhibits ccRCC cell migration and invasion. (A) The effect of PAPP‐A on the migration of ccRCC cells in the Transwell assay without Matrigel coating. (B) Matrigel‐coated Transwell assay was used to analyze the effect of PAPP‐A on ccRCC cell invasion. Scale bars: 1000 μm for low‐magnification images; 100 μm for high‐magnification images. *n* = 6. Mean ± SD is shown. **P < 0.01 by Student’s *t*‐test.

We performed Transwell assays with or without Matrigel coating to further investigate the function of PAPP‐A in ccRCC cell migration and invasion. Compared with control cells, ectopic expression of PAPP‐A significantly suppressed the migratory ability of 786‐O and 769‐P cells (Fig. 4A). The Matrigel Transwell invasion assay demonstrated that the invasive ability of 786‐O and 769‐P cells with enhanced expression of PAPP‐A was decreased compared with that of control cells (Fig. 4B).

### PAPP‐A regulates the expression of JNK and Wnt–β‐catenin pathway proteins

**Fig. 5:**
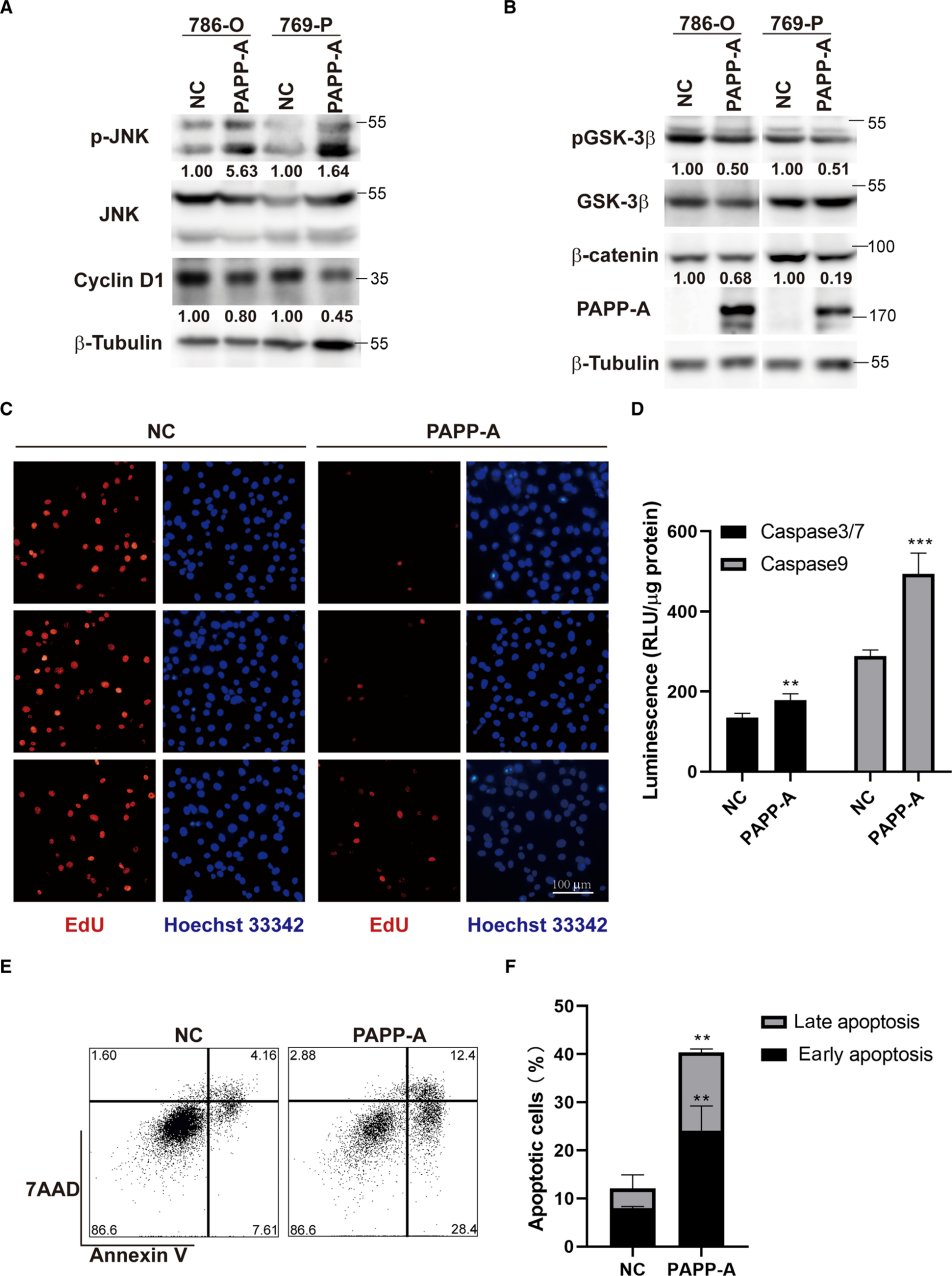
PAPP‐A promotes the JNK pathway and inhibits the Wnt–β‐catenin pathway in ccRCC cells. (A) The protein expression levels of p‐JNK, P21 and cyclin D1 were assessed in 786‐O and 769‐P cells transfected with PAPP‐A and control plasmid. (B) The expression levels of key proteins in the Wnt–β‐catenin signaling pathway were decreased in cells with high PAPP‐A expression. (C) Overexpression of PAPP‐A inhibits the EdU incorporation ability of ccRCC cells. (D) The cells transfected with PAPP‐A displayed higher caspase‐3/7/9 activity compared with control cells. RLU, Relative luciferase activity. (E, F) The cells transfected with PAPP‐A displayed higher frequency of apoptotic cells​, 7‐amino‐actinomycin D (7AAD) is a nuclear staining reagent. Scale bar: 100 μm. *n* = 3. Mean ± SD is shown. **P < 0.01, ***P < 0.001 by Student’s *t*‐test.

To determine the underlying mechanism of the regulatory effect of PAPP‐A on ccRCC cell proliferation, migration and invasion, we transfected ccRCC cells with PAPP‐A or control plasmid, and the expression levels of phosphorylated (p‐) JNK, JNK, cyclin D1, p‐GSK‐3β Ser9, GSK‐3β and β‐catenin were characterized by western blot. After we overexpressed PAPP‐A in 786‐O and 769‐P cells, the expression level of p‐JNK, an apoptosis‐related protein, was increased, indicating that PAPP‐A promotes the apoptosis of ccRCC cells. The expression of cyclin D1 decreased in PAPP‐A‐overexpressing cells (Fig. 5A). In addition, the expressions of key proteins of Wnt–β‐catenin signaling, p‐GSK‐3β Ser9 and β‐catenin were significantly decreased (Fig. 5B). Given that cyclin D1 and JNK pathways are critical regulators of cell cycle, cell proliferation and cell apoptosis, we further investigated the effect of PAPP‐A on EdU incorporation efficiency and cell apoptosis. Overexpression of PAPP‐A inhibits the cellular DNA replication in ccRCC cells as shown as the number of EdU‐positive cells in PAPP‐A‐transfected cells being significantly reduced as compared with the cells transfected with control plasmid (Fig. 5C). To examine the impact of PAPP‐A on cell apoptosis, we transfected ccRCC cells with PAPP‐A eukaryotic expression construct or with empty vector as a control. The cell apoptosis was characterized by measurement of caspase‐3/7/9 activity and flow cytometry. The cells transfected with PAPP‐A displayed significantly higher caspase‐3/7/9 activity (Fig. 5D) and higher frequency of apoptotic cells (Fig. 5E). The percentages of total apoptotic cells, early apoptotic cells and late apoptotic cells in the PAPP‐A‐overexpressed cells were 40.40% ± 4.73%, 24.13% ± 4.15% and 16.27% ± 0.58%, which were significantly higher than that of cells transfected with control vector (12.06% ± 2.61%, 7.99% ± 0.31% and 4.07% ± 2.30%, respectively) (Fig. 5F). All the results suggest that PAPP‐A promotes ccRCC cell apoptosis and suppresses RCC cell proliferation, migration and invasion through JNK and Wnt–β‐catenin pathways.

## Discussion

We herein demonstrate that PAPP‐A expression is decreased in ccRCC compared with normal controls. PAPP‐A is positively correlated with prognosis and is a prognostic factor for ccRCC. In addition, the survival time of grade 3 patients with low PAPP‐A expression is significantly shorter than that of patients with high PAPP‐A expression. Our results suggest that overexpression of PAPP‐A significantly affects the viability, proliferation, migration and invasion of ccRCC cells.

Pregnancy‐associated plasma protein A was first identified in the plasma of pregnant women, but its function was unknown [3]. PAPP‐A is located on chromosome 9q33.1, and its encoded protein belongs to the zinc‐binding metalloproteinase family, which affects IGF pathway activity through the cleavage of IGF binding proteins (IGFBPs) [18]. The cleavage of IGFBPs increases the levels of free IGF; IGF binds to IGF receptors and enhances IGF receptor signaling, which is associated with tumor growth, invasion and metastasis [19]. Subsequent studies revealed PAPP‐A to be ubiquitously expressed in a variety of normal cell types and tissues, and its levels are high in the kidney and bone [10, 11].

Previous studies have shown that PAPP‐A plays an important role in inflammation, bone formation and injury response [5, 20]. In addition, recent studies suggest that PAPP‐A acts as an oncogene in cancers, especially in breast cancer, ovarian cancer and lung cancer [21]. Pregnancy is associated with a temporally increased risk for breast cancer [22]. High PAPP‐A levels are maintained throughout pregnancy. PAPP‐A‐transgenic mice show increased proliferative signaling, indicating that PAPP‐A acts as a pregnancy‐dependent oncogene [23]. PAPP‐A can be detected in breast cancer tissues, and luminal B specimens have higher PAPP‐A expression, in terms of percentage of stained cells and intensity of staining, than luminal A specimens [24]. Most rodent breast cancer models and *in vitro* studies suggest that PAPP‐A is an oncogene in breast cancer that acts though the IGF signaling pathway [25, 26]. Another study found that a neutralizing monoclonal antibody of PAPP‐A not only repressed ovarian tumor growth but also reduced ascites accumulation and reversed platinum resistance [8]. Strikingly, the antitumor efficacy of the PAPP‐A antibody depends on the PAPP‐A expression level in tumors and ascites.

Conflicting with the aforementioned studies, several other studies point out the tumor suppressor properties of PAPP‐A. Loddo *et al*. [27] found that PAPP‐A is epigenetically silenced in precursor lesions and invasive breast cancer, and that downregulation of PAPP‐A promotes the invasion of breast cancer cells by regulating mitotic progression via modulating IGF‐1. Ectopic expression of STC2, an inhibitor of PAPP‐A, compromised breast cancer cell proliferation and motility [28]. However, high expression levels of STC2 were found in breast cancer tissues, especially in ER‐positive tissue [29]. As indicated earlier, several results conflict with the tumor‐promoting function of PAPP‐A. Further understanding of the expression level and molecular function of PAPP‐A in tumor progression needs to be performed. A detailed understanding of the function of PAPP‐A during tumor development and progression may account for conflicts such as these.

In this study, we first evaluated the expression level and function of PAPP‐A in RCC. Inconsistent with the other types of tumors, we found that PAPP‐A was barely expressed in RCC, whereas its expression was abundant in adjacent normal tissue. As mentioned earlier, in normal tissues, PAPP‐A expression levels are highest in the kidney and bone. We examined 85 cases of RCC and found that the mRNA and protein levels of PAPP‐A are very low in RCC tissue. This result indicated that PAPP‐A may act as a tumor suppressor in RCC. Further, we demonstrated that PAPP‐A expression inhibited cell viability, proliferation, migration and invasion.

According to previous research, the oncogenic function of PAPP‐A seems to be largely mediated by enhanced IGF signaling. We analyzed the IGF1 expression level in ccRCC using the TCGA database. There was no significant difference in the expression of IGF‐1 between normal and ccRCC tissues (Fig. S2A). The content of IGF‐1 in the supernatant of ccRCC cells and normal control cells (HK‐2) was measured by ELISA (4A Biotech), and we found that IGF‐1 content in the supernatant of ccRCC cells was lower than control cells (Fig. S2B). Besides, ELISA results showed that IGF‐1 was increased after overexpression of PAPP‐A, especially in 769‐P cells (Fig. S2C). Because IGF‐1 is well known as a tumor promoter, the current results appear to be conflicting with PAPP‐A tumor suppressor function. As discussed in the literature, the effect of PAPP‐A and IGF‐1 on atherosclerosis burden is also conflicting [30]. Overexpression of PAPP‐A increased the atherosclerosis burden, and IGF‐1 treatment reduced the atherosclerosis burden [31, 32]. It suggests that PAPP‐A may have biological effects by other means of functions independent of the IGF pathway. Moreover, TCGA database analysis results showed that IGF‐1 receptor expression is reduced in ccRCC tissue compared with normal tissue (Fig. S2D). Although the content of IGF‐1 is elevated in PAPP‐A‐overexpressed ccRCC cells, the decrease of IGF‐1 receptor may impair the function of IGF‐1. IGFBPs are the only known substrates of PAPP‐A proteolytic activity, but we still do not know if there are other unidentified substrates for PAPP‐A or if it has other biological activities unrelated to the IGF pathway. In this study, we also preliminarily investigated the molecular mechanism of PAPP‐A function in RCC and found that PAPP‐A overexpression increased the p‐JNK level, which induced more cell apoptosis. PAPP‐A suppresses cell proliferation by decreasing the expression of cyclin D1. Moreover, we showed that the expression of p‐GSK‐3β and β‐catenin decreased when PAPP‐A was overexpressed. The antimigration and anti‐invasion effects were likely associated with the Wnt signaling pathway, because p‐GSK‐3β and β‐catenin are two key factors in the Wnt pathway.

This study had several limitations. The regulatory mechanism of PAPP‐A expression in RCC is still elusive. A genome‐wide map of DNA copy number alterations and loss of heterozygosity (LOH) areas analysis results showed there was no statistical difference of LOH and copy number loss at 9q33.1 [33]. TCGA database analysis results indicate that the reduction in PAPP‐A mRNA expression may have no correction with DNA methylation level (Fig. S3A) [34]. EWS‐FLI‐1, also named EWSR‐1, binds to the PAPP‐A gene promoter and induces the expression of PAPP‐A in Ewing sarcoma [35]. We analyzed EWSR‐1 expression level in ccRCC using UALCAN [34]; EWSR‐1 expression is reduced in ccRCC (Fig. S3B), suggesting PAPP‐A may be regulated by EWSR‐1 in ccRCC. The earlier evidence suggested that PAPP‐A mRNA expression may regulate by EWSR‐1 or other unknown ways except DNA methylation, LOH and DNA copy number alterations. The possible mechanism still needs to be investigated in future studies. Furthermore, the detailed molecular mechanism involved in PAPP‐A‐mediated cell viability, proliferation, migration and invasion in RCC is also not clear. In addition, the function of PAPP‐A in RCC has been studied *in vitro*, but it remains to be determined whether it has a similar effect *in vivo*.

In addition, the results of this study are of great significance and application value. First, in contrast with other types of tumors, PAPP‐A expression was specifically lower in ccRCC than in adjacent normal tissue. This indicates that PAPP‐A may be a specific molecular marker for RCC. Although PAPP‐A is a secreted protein, its serum concentration does not seem to be a useful biomarker for ccRCC [36]. Because urine is closely associated with kidneys, further research should be done to investigate the PAPP‐A urine concentration in ccRCC and evaluate whether it is a useful factor for the early diagnosis or prognosis of ccRCC. Second, PAPP‐A expression appeared to suppress RCC cell growth, migration and invasion; therefore, it may be a target for clinical RCC therapy. Thus, further studies are ongoing to investigate the mechanisms involved in PAPP‐A‐mediated RCC development and progression, and to further elucidate the effect of PAPP‐A on RCC in animal models.

In conclusion, PAPP‐A is expressed at low levels in RCC and correlated with the worse outcome of RCC. Overexpression of PAPP‐A inhibits the proliferation, migration and invasion of RCC cells by activating JNK signaling and suppressing the Wnt–β‐catenin pathway.

## Conflicts of interest

The authors declare no conflict of interest.

## Author contributions

YL conceptualized the study, supervised the data, wrote the original draft and designed the methodology. SL provided resources and investigated the data. TW investigated and visualized the data. XL provided software and validated the data. LM provided resources. ZL supervised the data; administered the project; and wrote, reviewed, and edited the manuscript.

## Supporting information


**Fig. S1**. TCGA database analysis of tumor side and survival rate for localized and metastatic ccRCC by PAPP‐A expression.

**Fig. S2**. The expression level of IGF pathway in ccRCC tissue and cells.

**Fig. S3**. The possible regulatory mechanism of PAPP‐A reduction in ccRCC.

## Data Availability

The data that support the findings of this study are available in all figures and tables and the supplementary material of this article.
