# Supplementary figures and images for: PAPP‐A functions as a tumor suppressor and is downregulated in renal cell carcinoma
Source: FEBS Open Bio. 2021 May 2;11(6):1593–606. doi: 10.1002/2211-5463.13156 (PMC8167875; doi:10.1002/2211-5463.13156)

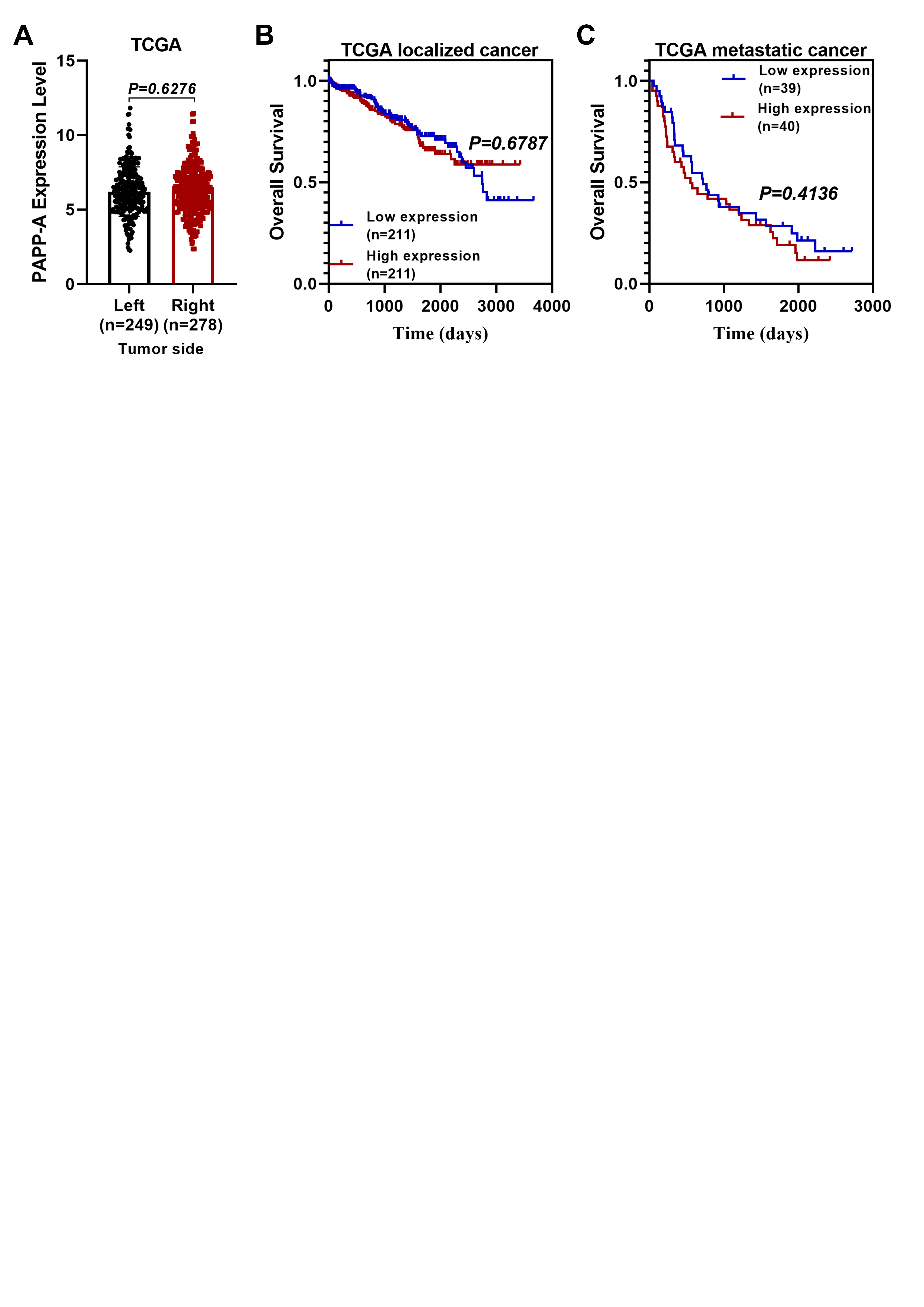

Supplement: Supplementary file 1 — Fig. S1. TCGA database analysis of tumor side and survival rate for localized and metastatic ccRCC by PAPP‐A expression. [file FEB4-11-1593-s002.tif]

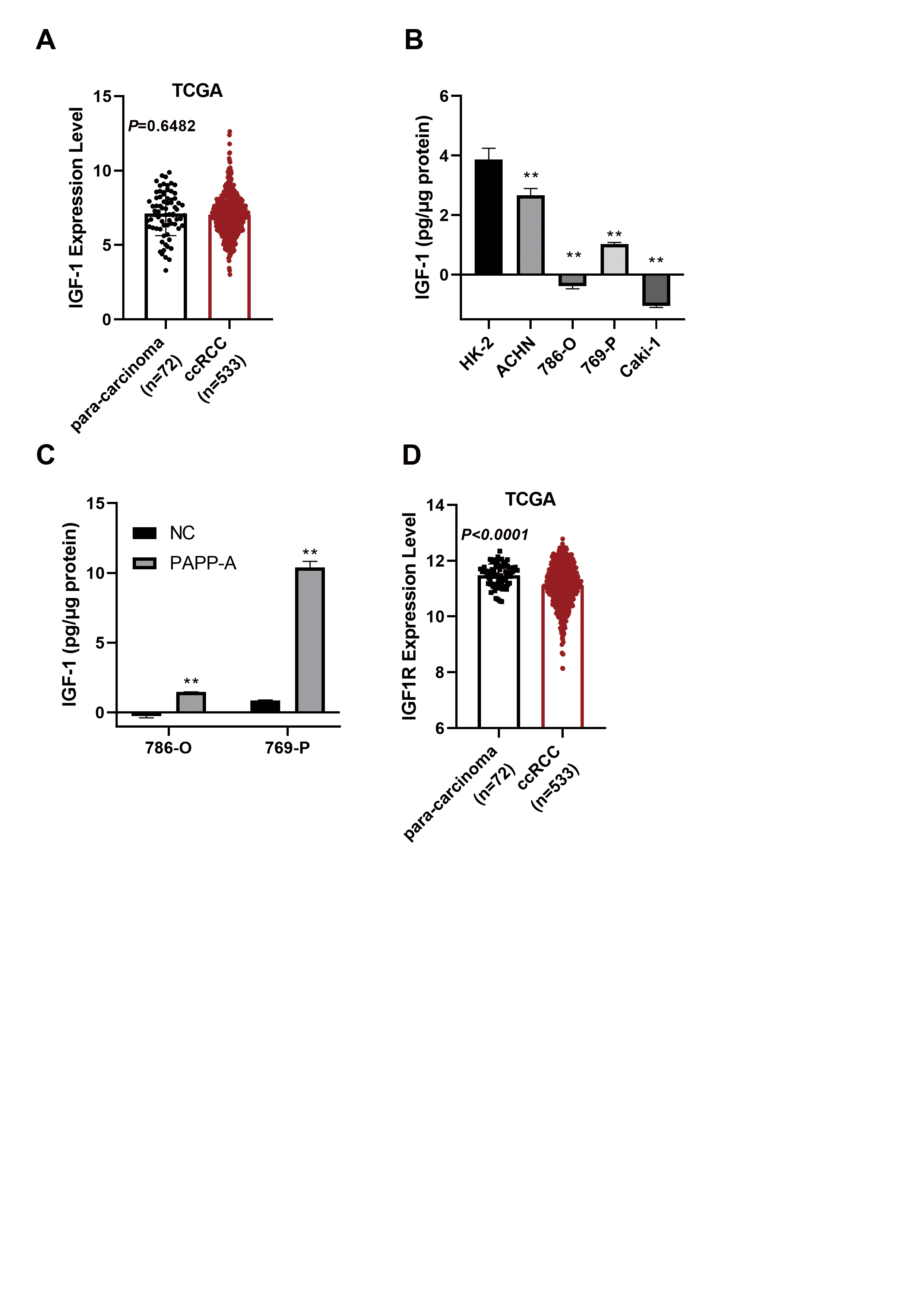

Supplement: Supplementary file 2 — Fig. S2. The expression level of IGF pathway in ccRCC tissue and cells. [file FEB4-11-1593-s004.tif]

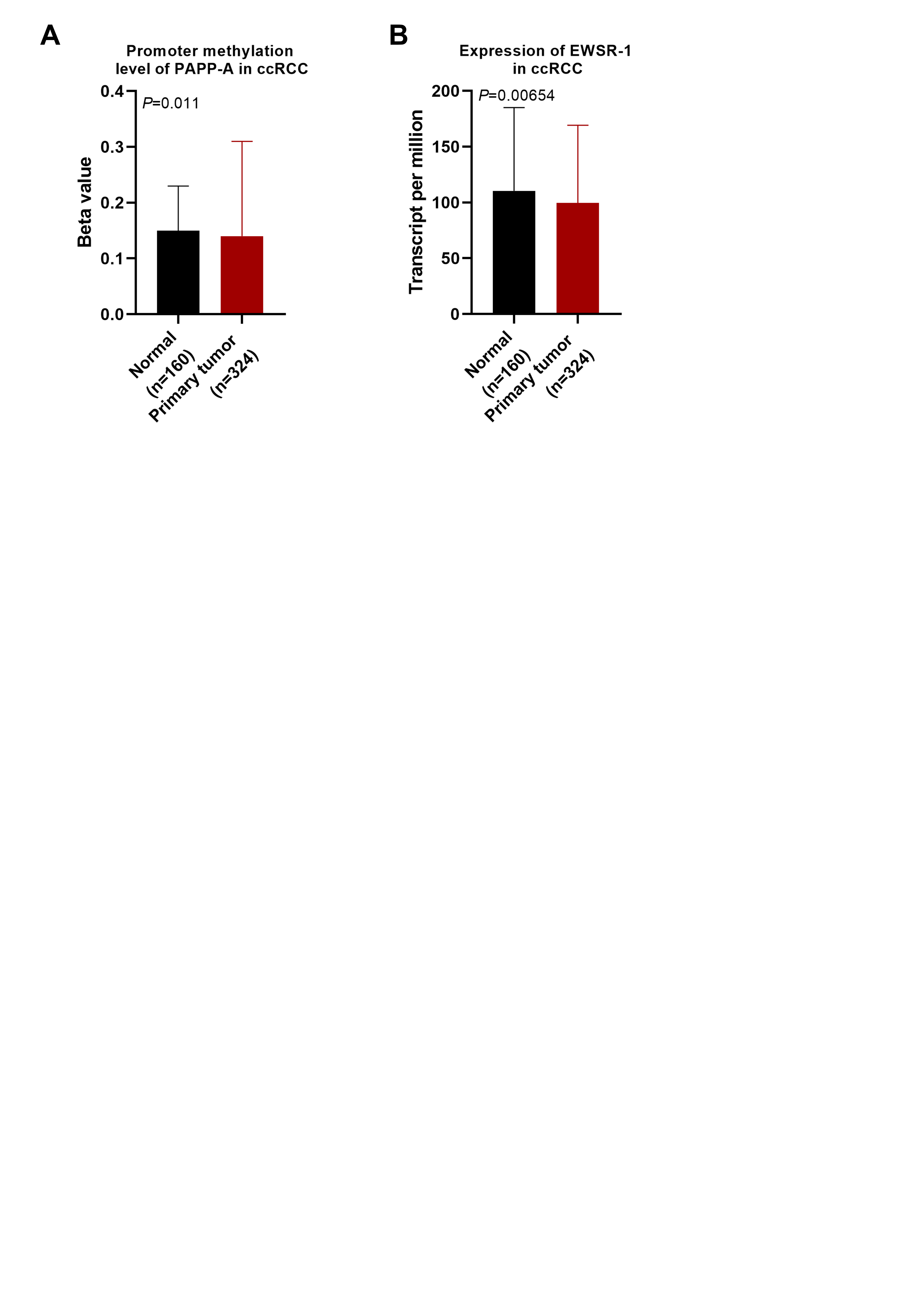

Supplement: Supplementary file 3 — Fig. S3. The possible regulatory mechanism of PAPP‐A reduction in ccRCC. [file FEB4-11-1593-s003.tif]
