# Supplementary material for: PAPP‐A functions as a tumor suppressor and is downregulated in renal cell carcinoma
Source: FEBS Open Bio. 2021 May 2;11(6):1593–606. doi: 10.1002/2211-5463.13156 (PMC8167875; doi:10.1002/2211-5463.13156)
Supplement: Supplementary file 4 [file FEB4-11-1593-s001.docx]

Figure S1 TCGA database analysis of tumor side and survival rate for localized and metastatic ccRCC by PAPP-A expression

A PAPP-A expression level and tumor side correction analysis in TCGA database (n=249, Left; n=278, Right). Mean±SD is shown. *P*=0.6276 by Student’s t-test. B&C TCGA analysis results showed PAPP-A expression has no significantly correction with the survival rate of localized and metastatic ccRCC. (n=211, localized cancer; n=39, n=40, metastatic cancer). *P* value by Log-rank text.

Figure S2 The expression level of IGF pathway in ccRCC tissue and cells

A TCGA analysis revealed that there was no significant difference in the expression of IGF-1 between normal and ccRCC tissues (n=72, para-carcinoma; n=533, ccRCC). Mean±SD is shown. *P*=0.6482 by Student’s t-test. B IGF-1 content in the supernatant of ccRCC cells was lower than control cells (n=3). Mean±SD is shown. **, *P*<0.01 by Student’s *t*-test. C PAPP-A increased IGF-1content in cell supernatant (n=3). Mean±SD is shown. **, *P*<0.01 by Student’s *t*-test. D TCGA analysis showed that IGF1R expression is reduced in ccRCC tissue compared with normal tissues (n=72, para-carcinoma; n=533, ccRCC). Mean±SD is shown. *P*<0.0001 by Student’s t-test.

Figure S3 The possible regulatory mechanism of PAPP-A reduction in ccRCC

A TCGA analysis revealed that promoter methylation olevel of PAPP-A was at low levels in ccRCC samples. B TCGA analysis results showed EWSR-1 expression was decreased in ccRCC compared with normal tissue. (n=160, Normal; n=324, Primary tumor). Mean±SD is shown. *P* value by Student’s t-test.
